# Assessment of the anti‐inflammatory and anti‐glycemic properties of Royal Jelly and Tocotrienol‐rich fraction in an experimental study: Does irisin mediate these effects?

**DOI:** 10.1002/fsn3.4321

**Published:** 2024-07-29

**Authors:** Naimeh Mesri Alamdari, Pardis Irandoost, Neda Roshanravan, Farzad Najafipour, Mohammadreza Vafa, Farnaz Farsi, Majid Mobasseri, Amir Ali Mir Mazhari, Halimeh AmirAzad, Farzad Shidfar

**Affiliations:** ^1^ Endocrine Research Center Tabriz University of Medical Sciences Tabriz Iran; ^2^ Department of Nutrition, School of Public Health Iran University of Medical Sciences Tehran Iran; ^3^ Cardiovascular Research Center Tabriz University of Medical Sciences Tabriz Iran; ^4^ Minimally Invasive Surgery Research Center Iran University of Medical Sciences Tehran Iran; ^5^ Department of Laboratory Sciences, Faculty of Para Medicine Tabriz University of Medical Sciences Tabriz Iran

**Keywords:** glucose hemostasis, inflammation, irisin, obesity, Royal Jelly, tocotrienol‐rich fraction

## Abstract

Irisin, a novel adipomyokine, has been proposed to be a therapeutic agent against obesity‐related metabolic disease. Royal Jelly (RJ) and tocotrienol‐rich fraction (TRF) are suggested to promote obesity and its related problems through potential mutual mechanistic pathways. This investigation intended to evaluate the glycemic and inflammation‐promoting effects of RJ, TRF, and their combinations to evaluate their synergic effects through irisin action in obese rats induced by a high‐fat diet (HFD) that underwent a calorie restriction diet (CRD). Fifty HFD‐fed obese rats received the following interventions: RJ, TRF, or RJ + TRF in combination with a CRD for eight consecutive weeks. After the investigation, body weight, fasting blood sugar (FBS), irisin, insulin, C‐reactive protein (CRP), interleukin‐6 (IL‐6), interleukin‐1 beta (IL‐1β), leptin, adiponectin, and insulin resistance (IR) were assessed. After 8 weeks of treatment, significant weight reduction was noticed in rats that received RJ and RJ + TRF related to the CRD rats (*p* < .001), although this reduction was not considerable in TRF‐treated rats. RJ and RJ + TRF supplementation markedly elevated irisin concentrations in CRD rats (*p* < .05), but TRF did not. Glycemic indices, inflammatory indices including IL‐1β and CRP levels, and leptin concentrations were significantly decreased after RJ, TRF, and their combinations were added to CRD (*p* < .05). According to the mediational analysis results, irisin mediated the promoting effects of RJ on glycemic hemostasis. Based on the results of this investigation, RJ and TRF are novel nutrients that have the potential to improve obesity‐related disorders. This research suggests that RJ exerts its beneficial glycemic regulatory effects through irisin.

## Graphical abstract

The important new findings of the present research are that Royal Jelly (RJ) remarkably elevates irisin concentrations in high‐fat diet (HFD)‐induced obese rats. Furthermore, irisin is probably the mediator of the promoting efficacy of RJ on glucose hemostasis in the obesity model. It is proposed that the effects of RJ and irisin on obesity‐induced metabolic disorders occur through common pathways, which include inducing thermogenesis through the browning of white adipose tissue (WAT), activation of brown adipose tissue, and increasing energy metabolism.

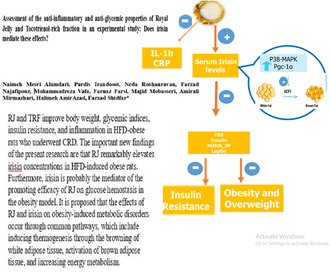

## 1 INTRODUCTION

Fatness is described by an extension of adipose tissue bulk, and it has been identified that adipose tissue impairment leads to the emersion of comorbidities of fatness, including glucose regulation disturbance, insulin resistance (IR), oxidative stress, and inflammatory state (Kinlen et al., 2018). Calorie restriction (CR) by creating a negative energy balance is one of the most frequently applied strategies for controlling overweight and obesity (Fontana & Klein, 2007).

Weight loss, which occurs in calorie restriction, decreases insulin resistance and ameliorates the metabolic disturbances related to obesity as a diabetes risk (Gabel et al., 2019). However, the efficacy of calorie restriction in the long term is limited (Liu et al., 2022). Therefore, there is an urgent necessity for new options in the fight against excessive body weight and associated complications.

Royal Jelly (RJ) as the product of worker bees is a natural adhesive milky material and has great features, such as hypoglycemic, antihypercholesterolemic, antitumor, anti‐inflammatory, hypotensive, antioxidant, antimicrobial, and antiaging characteristics, also promoting effects on atherosclerosis and infertility (Ahmad et al., 2020; Botezan et al., 2023; Pavel et al., 2011).

Royal Jelly (RJ) contains chief medium‐chain fatty acids, including trans‐10‐hydroxy‐2‐decenoic acid (10‐H2DA), 10‐hydroxydecanoic acid (10‐HDA), and sebacic acid (SEA), which are related to the health‐promoting characteristics of RJ. The proteins of RJ, such as major royal jelly protein 1 (MRJP1) and major royal jelly protein 3 (MRJP3), have important immune and anti‐inflammatory properties. Furthermore, the insulin‐like peptide contents of RJ decrease blood glucose levels and improve insulin resistance (Ramadan & Al‐Ghamdi, 2012; Vazhacharickal, 2021; Viuda‐Martos et al., 2008; Xue et al., 2017).

Recent investigations in animal models of obesity have revealed that RJ combats obesity and related comorbidities by increasing thermogenesis and producing beige adipose tissue (white fat browning effect) by upregulating thermogenic genes, including uncoupling protein 1 (UCP‐1) and other downstream transcription factors, like PR domain containing 16 (PRDM‐16), p38 mitogen‐activated protein kinases (p38 MAPK), and bone morphogenetic protein 8B (BMP8B) (Mesri Alamdari et al., 2020; Yoneshiro et al., 2018).

Tocotrienols (T3s), as subcategories of vitamin E, have an unsaturated side chain, and in the net form and complex form with other T3 isomers, they possess potent inflammation‐promoting, cardioprotective, diabetes‐improving, and cancer‐protective characteristics in human and experimental studies (Choi & Lee, 2009; Pang et al., 2023; Pang & Chin, 2019; Peh et al., 2016; Sadikan et al., 2022; Wong & Radhakrishnan, 2012).

It is suggested that T3s have promising anti‐obesity properties. Recently, numerous investigations have propounded the anti‐obesity mechanisms of T3s, including reducing the adiposity and differentiation of adipocytes, effects on energy sensing and energy expenditure, impact on apoptosis of preadipocytes, and modifying inflammation (Tanaka‐Yachi et al., 2022; Zhang et al., 2021; Zhao et al., 2016). In obese animals, γ‐tocotrienols (γT3s) diminished fasting blood glucose, insulin, body weight, and pro‐inflammatory cytokines (Zhao et al., 2015). Furthermore, T3s boost the messenger RNA (mRNA) rate of peroxisome proliferator‐activated receptor gamma coactivator 1‐alpha (PGC‐1α) and uncoupling protein 1 (UCP‐1) in adipocytes, which all contribute to the increase in thermogenesis (Tanaka‐Yachi et al., 2018).

Currently, the mutual collaboration of adipose and muscle tissues is identified to have a vital role in body weight modulation. Adipose and muscle tissues, through secretion of some cytokines, adipocytes, and myokines, make tissue connections and establish metabolic homeostasis (Gallagher et al., 2005; Kokta et al., 2004).

Irisin is the product of the Fndc5 gene and mostly diffuses through muscle, subcutaneous white adipocytes, and somewhat from visceral adipocytes in response to exercise, feeding state, and critical states, such as obesity and anorexia (Raschke et al., 2013).

It is proposed that irisin, which is regulated by the peroxisome proliferator‐activated receptor gamma coactivator 1‐alpha (PGC‐1α) gene, encodes a thermogenic protein. It enhances energy expenditure and white adipose tissue (WAT) beigeing by inducing UCP‐1 mRNA levels (Arhire et al., 2019; Hofmann et al., 2014).

Beyond the browning effect of irisin, it ameliorates glucose metabolism by accelerating glucose uptake in skeletal muscles. It is conceived that irisin affects organs that have a pivotal role in type 2 diabetes, including the liver and pancreas, through decreasing insulin resistance and ameliorating glucose and lipid metabolism. Therefore, it operates as an insulin‐sensitizing hormone (Perakakis et al., 2017; Polyzos et al., 2018). Considering the health‐promoting impacts of irisin, it might be a novel approach to combating obesity and its complications.

The promoting effects of RJ and tocotrienol‐rich fraction (TRF) on metabolic regulation through irisin on high‐fat diet‐induced obesity model were evaluated by our research group. The results of the current study revealed that glucose homeostasis and inflammation, which were mediated by irisin, were improved when RJ and TRF were added to high‐fat diet obese rats. Furthermore, RJ increased the irisin concentration, but TRF did not have remarkable effects on irisin levels (Irandoost, Mesri Alamdari, Saidpour, Shidfar, Roshanravan, et al., 2020). Although, in a recent study 350 mg/kg dose of RJ by gavage in diabetic rats decreased blood glucose but did not significantly increase irisin levels (Çakır, 2023).

Regarding the anti‐obesity characteristics of RJ and TRF and the common regulatory metabolic pathways of RJ, TRF, and irisin, we speculated that RJ and TRF, as functional food substances, might act through irisin induction with mutual mechanistic pathways. Therefore, we aimed to assess the effects of RJ, TRF, and their combinations as synergic effects of them on glucose hemostasis and inflammatory indices and the intermediary role of the irisin in the positive role of RJ and TRF as the functional food in obese rats fed a calorie‐restricted diet.

## 2 MATERIALS AND METHODS

### 2.1 Animals and interventions

Fifty, 3‐week‐old male Wistar rats in the weight range of 50–70 g were obtained from Pasteur Institute (Tehran, Iran) and housed separately in a room at 22 ± 2°C with 50 ± 10% humidity and a 12 h light, dark cycle.

The Ethics Committee of Iran University of Medical Sciences (ethic code: IR.IUMS.FMD. REC 1396.9321324003) authorized the animal experimental procedures that were carried out according to the National Institutes of Health Guide for the Care and Use of Laboratory Animals (National Research Council, 2010) and the ARRIVE (Animal Research: Reporting of In Vivo Experiments) guidelines. We tried to decrease the number of rats and their suffering.

At 4 weeks of age, an HFD was given to all rats to induce obesity. Tap water and ad libitum food were provided freely for rats.

The HFD includes standard normal diet (chow powder) blended with milk butter (40% w/w). Approximately, 60% of total calorie of diet was provided by fat. Table 1 indicates the components of the diets applied in this investigation. The composition of chow diet was present with the company (Behparvar, manufacturer of feed, supplements, and concentrates for livestock, poultry, and aquatic animals, Iran).

**TABLE 1:** Components of applied diets.

Dietary composition (g/kg)	ND	HFD
Carbohydrate	536.2	335.1
Fiber	42	26.2
Protein	260.8	163
Lipid	40	400
Calcium	9.5	5.93
Phosphorus	6.5	4.06
Salt	5	3.12
Moisture	50	31.2
Ash	50	31.2
Energy density (kcal/g)	3.6	5.6

Abbreviations: HFD, high‐fat diet; ND, normal diet.

The obesity in rats was improved when the average weight of HFD‐prescribed rats elevated markedly relative to rats with normal chow diet intake at the 17‐week age of rats. Five rats received normal diet (chow diet) and were considered as control group for HFD receiving rats.

After 17 weeks, the average weight of HFD receiving rats increased remarkably relative to normal group rats (443.28 g ± 46.62 g vs. 396.24 g ± 28.79 g *p* < .05), which confirmed the HFD model was achieved. Furthermore, the body weight and body length were used to determine the following anthropometric parameters: Body mass index (BMI): body weight (g)/length^2^ (cm^2^) and those with BMI values ≥30 were considered obese (Novelli et al., 2007). After the obesity induction phase, five control rats receiving normal (chow) diet were excluded from the study. They were not included in the next phase of study.

**FIGURE 1:**
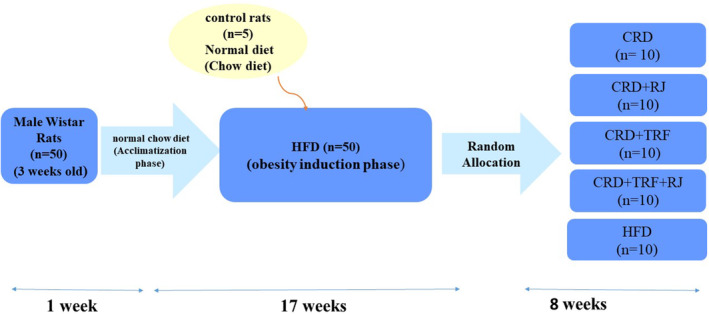
Study protocol.

Figure 1 shows the study protocol in detail. At the next phase, 50 obese rats were randomly allocated to five groups (*n* = 10/group) and cured for eight consecutive weeks: RJ group, received 100 mg/kg/day RJ powder and CRD, TRF group, received 85 mg/kg/day TRF and CRD, RJ + TRF group, simultaneously received 100 mg/kg/day RJ powder and 85 mg/kg/day TRF, CRD group, received CRD as the control for treatments, and HFD group, received HFD as the control for CRD.

The supplement amounts and period of the investigation were defined based on the safety reports of previous studies (Wong et al., 2017; Zamami et al., 2008). CRD and HFD are the same in terms of content, except for the calorie amount, which was 30% below the HFD (Liu et al., 2022; Speakman & Mitchell, 2011). The treatments were appended to the CRD, and prepared foods were weighed daily and administered every 9:00 am. Furthermore, an HFD was provided ad libitum for rats daily.

Royal Jelly (RJ) powder was prepared from the Bulk Supplements Company (Henderson, USA) in the lyophilized form and contains 6% 10‐HDA. The TRF supplement was awarded from the ExcelVite Sdn. Bhd. (Perak, Malaysia) company and contains the following tocotrienol and tocopherol isomers: 12% α‐tocotrienol, 2% β‐tocotrienol, 19.3% γ‐tocotrienol, and 5.5% δ‐tocotrienol (5.5%) and 11.9% total α‐tocopherol.

After 8 weeks of intervention, rats were kept fasting for 12 h and were anesthetized with an intraperitoneal injection of 20 mg/mL xylazine 2% (Alfasan, Netherlands) and 100 mg/mL ketamine 10% (Alfasan, Netherlands). Blood was gathered through cardiac puncture and centrifuged at 3000 *g* for 15 min at 25°C. Serum samples were isolated and stockpiled in −80°C until examinations.

Fasting blood sugar (FBS) was determined enzymatically. The serum irisin, insulin, C‐reactive protein (CRP), interleukin‐6 (IL‐6), interleukin‐1 beta (IL‐1β), and adipokine concentrations were determined using kits (MyBioSource, Inc., San Diego, USA) based on the kit protocol and enzyme‐linked immunosorbent assay (ELISA) method. IR was determined via the Homeostatic Model Assessment of Insulin Resistance (HOMA‐IR) method. Fasting glucose (milligrams per deciliter (mg/dL)) × fasting insulin (micro‐international units per milliliter (μIU/mL))/405 was used to measure the HOMA‐IR.

### 2.2 Statistical analysis

The normality of data was evaluated through one‐sample Kolmogorov–Smirnov test. Results were presented as the mean ± SEM. One‐way analysis of variance (ANOVA) and Tukey's post hoc test were applied to compare between groups. Serum irisin levels correlated with glucose hemostasis and inflammatory markers were calculated by Pearson's correlation analysis. Analysis of covariance (ANCOVA) was applied to adjust the effects of irisin on the between‐groups comparison. SPSS Statistics (version 26) was used to analyze the data. GraphPad Prism (version 9) was used to draw the figures. The level of significance was regarded as *p*‐value <.05.

## 3 RESULTS

### 3.1 The effects of CRD relative to HFD on weight, irisin, and biochemical index levels of obese rats

**FIGURE 2:**
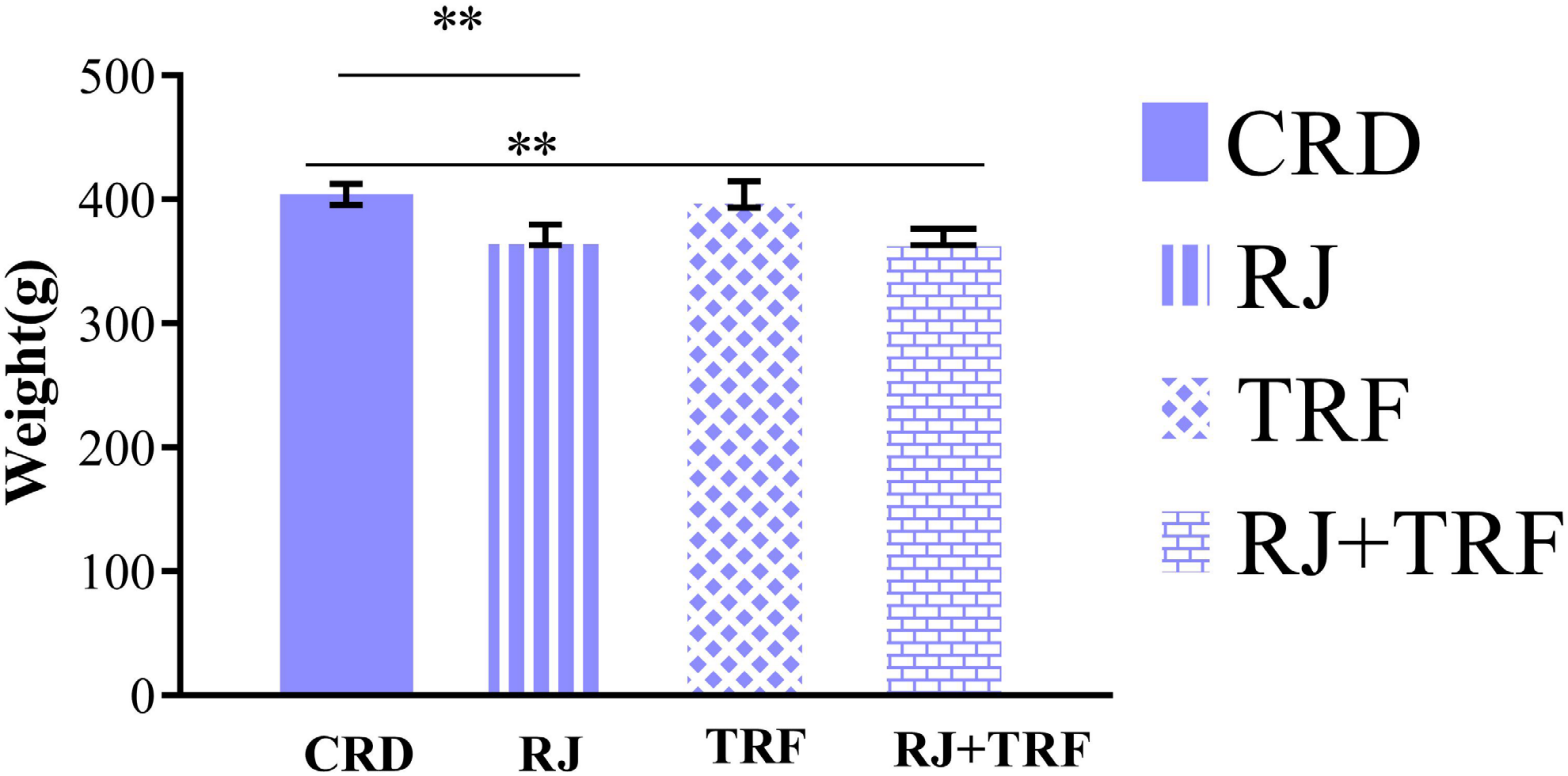
Comparison of (a) weight, (b) serum irisin levels, (c) glycemic indices, (d) inflammatory indices, and (e) adipokine concentrations in the calorie‐restricted diet (CRD) group vs. high‐fat diet (HFD) groups; data are presented as the mean ± SEM; **p < .010, **p < .001.

Eight weeks of CRD in obese rats led to a remarkable decrement in weight in comparison to that of the HFD‐induced obese rats (*p* < .001) (Figure 2a). As shown in Figure 2b, CRD decreased the serum levels of irisin, albeit not significantly (*p* = .201). Glucose hemostasis indices, such as FBS (*p* = .021), insulin (*p* = .017), HOMA‐IR (*p* = .017), IL‐1β (*p* = .001), IL‐6 (*p* = .003), CRP (*p* = .001), and leptin (*p* = .024), declined significantly following CRD in obese rats relative to the HFD rats (Figure 2c–e). Furthermore, adiponectin levels improved insignificantly in CRD rats (*p* = .071) (Figure 2e).

### 3.2 The effects of Royal Jelly, TRF, and their combinations with CRD on weight, irisin, and biochemical index levels of obese rats

**FIGURE 3:**
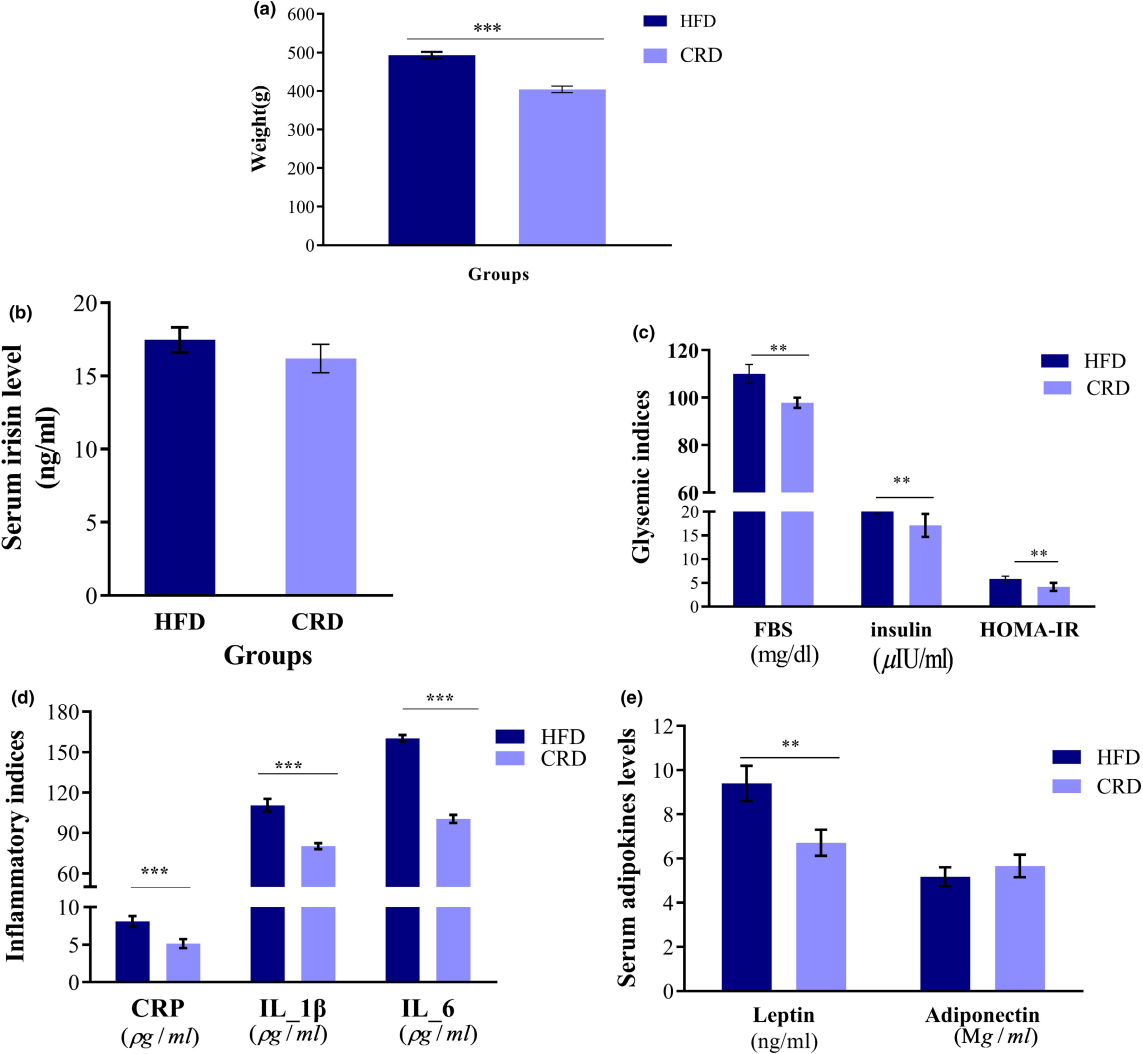
Comparison of weight in the RJ, TRF, and RJ + TRF groups relative to the CRD groups; data are presented as the mean ± SEM; **p < .010, **p < .001.

As presented in Figure 3, 8 weeks of treatment significantly reduced the weight of RJ‐ and its combination with TRF‐treated rats in comparison to the CRD‐treated rats (*p* < .001), although this reduction was not considerable in the TRF group. As tabulated in Table 2, RJ and its combination with TRF treatments, but not TRF alone, markedly elevated irisin concentrations in CRD rats, respectively (*p* = .033, *p* = .019). FBS, insulin, and HOMA‐IR were significantly decreased after RJ, TRF, and their combinations were added to the CRD. RJ, TRF, and their combinations led to a marked improvement in inflammatory parameters, including IL‐1β and CRP levels, in CRD rats, although IL‐6 levels did not change remarkably. Leptin concentrations decreased notably in RJ and RJ + TRF supplemented rats relative to the CRD rats but not in the TRF‐treated rats. RJ and TRF did not change the adiponectin levels significantly.

**TABLE 2:** Comparison of biochemical parameters between studied groups after 8 weeks of intervention.

Variables	RJ	TRF	RJ + TRF	CRD	*p*‐Value^a^
Irisin (ng/mL)	19.53 ± 0.84	17.87 ± 0.68	19.78 ± 0.75	16.19 ± 0.97	
*p*‐Value^b^	.033	.481	.019		.013
FBS (mg/dL)	88.91 ± 1.94	82.44 ± 1.46	83.73 ± 1.74	97.79 ± 2.18	
*p*‐Value^b^	.009	<.001	<.001		<.001
*p*‐Value^c^	.095	<.001	.005		<.001
Insulin (μIU/mL)	12.32 ± 0.32	14.34 ± 0.52	12.15 ± 0.45	17.13 ± 0.41	
*p*‐Value^b^	<.001	<.001	<.001		<.001
*p*‐Value^c^	.003	<.001	.002		<.001
HOMA‐IR	2.71 ± 0.11	2.92 ± 0.14	2.52 ± 0.13	4.15 ± 0.18	
*p*‐Value^b^	<.001	<.001	<.001		<.001
*p*‐Value^c^	.001	<.001	.004		<.001
Leptin (ng/mL)	4.87 ± 0.41	6.57 ± 0.22	4.71 ± 0.39	6.71 ± 0.59	
*p*‐Value^b^	.022	.996	.011		.002
*p*‐Value^c^	.083	.69	.060		.050
Adiponectin (pg/mL)	6.90 ± 0.60	6.03 ± 0.53	6.60 ± 0.50	5.66 ± 0.51	
*p*‐Value^b^	.384	.963	.617		.381
*p*‐Value^c^	.623	.942	.968		.929
CRP (pg/mL)	3.42 ± 0.36	3.06 ± 0.42	3.16 ± 0.25	5.14 ± 0.60	
*p*‐Value^b^	.036	.008	.013		.005
*p*‐Value^b^	.013	.003	.010		.015
IL‐1β (pg/mL)	75.52 ± 0.47	74.68 ± 0.58	73.94 ± 0.60	80.21 ± 2.23	
*p*‐Value^b^	.047	.014	.005		.004
*p*‐Value^c^	.041	.006	.007		.021
IL‐6 (pg/mL)	92.65 ± 1.99	94.78 ± 1.96	90.55 ± 2.85	100.43 ± 3.01	
*p*‐Value^b^	.143	.394	.040		.049
*p*‐Value^c^	.003	.031	.001		.006

*Note*: Data are presented as the mean ± SEM (*n* = 10).

Abbreviations: CRD, calorie restriction diet; CRP, C‐reactive protein; FBS, fasting blood sugar; HOMA‐IR, Homeostatic Model Assessment of Insulin Resistance; IL‐1β, interleukin 1 beta; IL‐6, interleukin 6; RJ, Royal Jelly; TRF, tocotrienol‐rich fraction.

^a^ *p*‐Value is based on between‐groups comparison by one‐way ANOVA.

^b^ *p*‐Value is based on comparison vs. CRD group by one‐way ANOVA.

^c^ *p*‐Value is based on the ANCOVA test adjusted for irisin.

### 3.3 Assessment of the correlation of irisin concentration with glucose hemostasis indices and inflammatory markers in CRD obese rats

As reported in Table 3, serum irisin concentration correlates negatively with FBS, insulin, HOMA‐IR, and leptin levels in CRD obese rats (*p* < .001). However, it was not significantly associated with inflammatory indices. Moreover, irisin concentration correlated positively with adiponectin concentration based on Pearson's correlation test (*p* < .001).

**TABLE 3:** Association of serum irisin values (ng/mL) with glycemic, inflammatory, and adipokine levels.

	FBS	Insulin	HOMA‐IR	CRP	IL‐6	IL‐1β	Leptin	Adiponectin
Irisin	*n* = 40							
*r*	−.630	−.707	−.739	−.252	−.326	−.326	−.572	.453
*p*‐Value	<.001	<.001	<.001	.117	.597	.050	<.001	<.001

*Note*: *p*‐Value and *r* are calculated through Pearson's correlation analysis.

Abbreviations: CRP, C‐reactive protein; FBS, fasting blood sugar; HOMA‐IR, Homeostatic Model Assessment of Insulin Resistance; IL‐1β, interleukin 1 beta; IL‐6, interleukin 6; RJ, Royal Jelly; TRF, tocotrienol‐rich fraction.

### 3.4 Assessment of the mediatory effects of irisin

We conducted a mediational analysis to survey whether irisin might be the connector molecule through which RJ and TRF affect glycemic and inflammatory markers. To test the irisin mediatory effect hypothesis, the Baron and Kenny approach was used (1986). According to Baron and Kenny's approach, irisin is recognized as the mediator of the effects of RJ on glycemic indices and leptin because (1) RJ is associated with changes in glucose hemostasis markers, including FBS, insulin, HOMA‐IR, and leptin levels (Table 2). (2) RJ is associated with changes in irisin levels as hypothesized mediators (Table 2). (3) Changes in irisin concentrations are related to changes in FBS, insulin, HOMA‐IR, and leptin levels after controlling for RJ (Table 3). (4) A previously significant association between RJ and FBS, insulin, HOMA‐IR, and leptin was attenuated or no longer significant when controlling for the effect of irisin on the above‐mentioned outcomes (Table 2). According to the mediational analysis results, irisin is not considered the mediator of the effects of RJ on inflammatory markers and adiponectin. Regarding the effects of TRF on glycemic and inflammatory indices, irisin does not mediate the anti‐glycemic and anti‐inflammatory effects.

## 4 DISCUSSION

Obesity is now considered a pandemic problem. Regarding the poor efficacy of calorie restriction diets in obesity management, the application of adjunctive therapeutic approaches that reduce body weight and counteract the comorbidities of obesity is noteworthy.

We demonstrate in the present investigation that the application of calorie restriction in obese rats diminished body weight and improved glucose, insulin, HOMA‐IR, leptin, and inflammation. Furthermore, the decrease in circulating irisin levels following CRD intervention in HFD‐induced obese rats, which was approved by previous animal and clinical trials, indicated that weight loss induced by a weight‐reduction program and bariatric surgery reduced circulating irisin levels (Cao et al., 2019). However, there are inconsistencies in the correlations of irisin levels with overweight and obesity (Arhire et al., 2019; Cao et al., 2019). Some claimed that irisin declined following obesity, and others suggested an increase in irisin levels following adiposity (Huh et al., 2012; Polyzos et al., 2018). An increase in irisin concentrations in an obese state for a long period is a pathological condition and might be an adaptive compensatory response to hyperglycemia, although it finally results in potential irisin resistance, such as insulin and leptin resistance, which occurs in obesity (Yan et al., 2014).

Royal Jelly (RJ) and TRF, acting as novel functional foods, provide health benefits beyond their ordinary function (Guo et al., 2021). The authors reported in a recent investigation that RJ is capable of inducing thermogenesis by increasing the expression of thermogenic factors, including UCP‐1, PRDM‐16, p38 MAPK, and other downstream genes, which gave rise to the remodeling of white fat and the activation of classical brown fat in an obesity model of HFD‐induced rats (Mesri Alamdari et al., 2020).

The findings of the present investigations showed that RJ and its combination with TRF treatment in obese rats that underwent CRD decreased body weight remarkably relative to CRD alone. However, TRF did not change the body weights of obese rats that received CRD in the current investigation. This is in agreement with the results of the Wong et al. (2012) and Yoneshiro et al. (2018). Furthermore, according to the findings of our study, adding the RJ, TRF, and their combinations to obese rats that underwent CRD ameliorated irisin, FBS, insulin concentration, and HOMA‐IR. A high‐fat diet contributes to metabolic dysfunction of adipose tissue, including insulin resistance, along with the toxic effects of fat depots on pancreatic beta cells (β cells) (Chalkley et al., 2002). Although the exact mechanisms of the relationship between increased body fat and insulin resistance are not fully understood, it is proposed that enhanced amounts of free fatty acids play an important role by disturbing the glycolytic pathway and impairing glucose attraction to the cells, utilization, and oxidation in tissues that are sensitive to insulin (Pratchayasakul et al., 2011; Silveira et al., 2008).

The beneficial health‐promoting actions of RJ and TRF supplementation on the regulation of glucose and insulin resistance have been confirmed in previous studies (Chia et al., 2016; Chung et al., 2019; Ibrahim & Kosba, 2018). However, in the present investigation, we have emphasized the role of white and brown adipose tissue in glucose hemostasis. We previously reported that receiving 100 mg RJ/kg/day by CRD obese rats enhanced the induction of UCP‐1 in white and brown adipose tissue and decreased the body weights of the rats without any change in food intake. We have suggested that the glycemic and insulin resistance control of RJ is accomplished through inducing thermogenesis and the production of beige adipocytes (Mesri Alamdari et al., 2020).

Similar to our results, Yoneshiro et al. (2018) reported that RJ improves glucose hemostasis by activating brown adipose tissue, increasing thermogenesis, and inducing UCP‐1, PGC‐1α, and mitochondrial biogenesis.

Regarding the effects of irisin on thermogenesis, we speculated in a previous study that irisin is a signaling molecule through which RJ and TRF act. Interestingly, we observed that 8 weeks of RJ treatment and RJ in combination with TRF in high‐fat diet‐induced obese rats resulted in the elevation of irisin values, and negative correlations of irisin levels with glycemic indices were demonstrated (Irandoost, Mesri Alamdari, Saidpour, Shidfar, Roshanravan, et al., 2020). It is the first report to evaluate the effect of RJ and TRF as functional foods on obesity problems through irisin mediation. We reported that that RJ might increase the irisin secretion through PGC‐1α activation, which subsequently regulates the glycemic and inflammatory pathways, and results in antihyperglycemic and anti‐inflammatory effects (Irandoost, Mesri Alamdari, Saidpour, Shidfar, Roshanravan, et al., 2020).

In the recent study by Selcen ÇAKIR, 350 mg/kg dose of RJ by gavage in diabetic rats for 4 weeks decreased blood glucose but did not significantly increase irisin levels. The reason for this may be associated with the design of study which was focused on diabetic rats, and the duration of administration of which was 4 weeks (Çakır, 2023). It is reported that, first, irisin increases in serum of the obese and diabetic groups and in the second stage, this mechanism is depleted or acclimated, possibly resulting in lower irisin secretion.

It is suggested that FNDC5's (fibronectin domain‐containing protein 5) ectodomain was cleaved by a protease, gave rise to the soluble irisin protein, and traveled by the blood to the fat tissue with transcription factors, and finally transformed the white or beige adipocyte into the brown named as the browning effect (Erickson, 2013).

We conducted a mediational analysis to investigate whether irisin mediates the anti‐glycemic role of RJ and TRF. According to the results of the present study, irisin acts as a mediator of the improvement in FBS and insulin resistance following RJ treatment in obese rats. However, TRF did not present any promising impact on circulating irisin values.

It is suggested that irisin may ameliorate glucose attraction to cells and improve insulin resistance by the p38 MAPK–PGC‐1α pathway. Thus, the useful metabolic regulatory properties of RJ mediated through irisin include effects on thermogenesis, an increase in the activity of brown adipose tissue and the production of beige adipose tissue, the induction of the p38 MAPK and PGC‐1α, translocation of glucose transporter type 4 (GLUT4), and the facilitation of glucose uptake in cells (Ye et al., 2019).

The present investigation failed to demonstrate the mediatory action of irisin on the anti‐glycemic effects of TRF. Therefore, the suggested mechanism of the anti‐glycemic functions of TRF is increasing insulin sensitivity by the insulin signaling route and phosphorylation of insulin substrate receptor 1 (IRS‐1) and protein kinase B (Akt), the ligand role of TRF on the transcription factors such as peroxisome proliferator‐activated receptor gamma (PPAR‐γ) and peroxisome proliferator‐activated receptor delta (PPAR‐δ), which are important regulatory factors of glucose and lipid metabolism, effects on the (AMPK)/sirtuin 1 (SIRT1)/PGC‐1α energy metabolism pathway in muscle cells and inducing mitochondrial biogenesis, induction of GLUT4 in muscle cells, and facilitation of glucose uptake (Chia et al., 2016; Mahjabeen et al., 2021; Phang et al., 2023; Wong et al., 2012; Zhao et al., 2015).

Royal Jelly (RJ), TRF, and their combinations contribute to a remarkable decrement of inflammatory markers, including CRP and IL‐1β. According to the four steps of Barron and Kenny's mediational analysis results, irisin does not present a mediatory function in the inflammation‐inhibiting impacts of RJ and TRF. Therefore, the suppressive effects of RJ and TRF on inflammation are not attributed to irisin.

The results of the present study are in line with the results of a study by Petelin et al. (2019), which reported the promising effects of RJ on CRP and adiponectin levels in overweight adults. They suggested that an induced expression of adiponectin and adiponectin receptor 1 (AdipoR1) along with the induced phosphorylated AMP‐activated protein kinase (pAMPK) and peroxisome proliferator‐activated receptor‐α (PPAR‐α) reduces inflammation and oxidative stress following RJ treatment.

Furthermore, Almeer et al. (2018) revealed that administration of RJ prompted a noticeable reduction in IL‐1β and TNF‐α concentrations in mice that have hepatotoxicity. They suggested that three main fatty acids, including 10‐H2DA, 10‐HDA, and sebacic acid (SEA), attenuated inflammation via inhibition of the mRNA levels of pro‐inflammatory cytokines and suppression of NF‐κB (nuclear factor‐kappa B) translocation. RJ prevents the generation of pro‐inflammatory cytokines, such as TNF‐α, IL‐6, and IL‐1β, through the major royal jelly protein 3 (MRJP3), which belongs to the major royal jelly protein (MRJP) class and is responsible for 82%–90% of total RJ proteins (Zamami et al., 2008). Additionally, the most important proposed mechanisms for the inflammation‐suppressive effects of T_3_ are the inhibition of NF‐κβ, c‐Jun N‐terminal kinase (JNK), and extracellular singal‐regulated kinase (ERK) passage. Moreover, the antioxidant effects of T_3_ are another prominent factor in the suppression of inflammatory mediators (Irandoost, Mesri Alamdari, Saidpour, Shidfar, Farsi, et al., 2020; Khor et al., 2021).

Adiponectin is an insulin‐sensitizing adipocyte with diabetes‐preventive properties, and a decrease in adiponectin values results in adiposity and insulin resistance. In this study, RJ and TRF resulted in an insignificant increase in adiponectin levels, which is in agreement with the Yoshida et al. (2017) investigation, which demonstrated that 10 mg/kg/day RJ in Kuo‐Kondo–Agouti yellow (KK–Ay) obese rats with diabetes for 4 weeks through oral gavage caused insignificant enhancement of adiponectin levels and remarkable adiponectin mRNA and adiponectin receptor AdipoR1 expression. Increased expression levels of adiponectin mRNA and its receptor as an insulin sensitivity agent, followed by an enhancement of the pump mRNA levels and PGC‐1α, contribute to glycemic and insulin resistance control. Matsunga et al. reported that treatments of T3‐L13 inflammatory adipocytes with γT3 contribute to the induction and secretion of adiponectin. Researchers have suggested that γT3, through inhibition of NF‐κB, results in the promotion of an inflammatory state and recovery of adiponectin levels (Matsunaga et al., 2012). In the present investigation, although RJ and TRF gave rise to an insignificant increase in adiponectin values, it might be significant if the doses of RJ and TRF were higher or if the duration of the study was longer.

In total, our investigation suggests that RJ and TRF improve body weight and obesity‐related comorbidities, including inflammation and hyperglycemia, in obese rats, and amplify the beneficial impacts of a calorie‐restricted diet. Furthermore, we demonstrated that the hypoglycemic properties of RJ are related to irisin since it mediates the antihyperglycemic effects of RJ. RJ contributes to irisin increment, which consequently induces the genes involved in thermogenesis, such as p38 MAPK, UCP‐1, and PGC‐1α, and the development of white adipose tissue browning, promoting the energy expenditures that give rise to glucose hemostasis progression and insulin resistance. To the best of our knowledge, this is a pioneering investigation that evaluates the mediatory function of irisin and its effect on the glycemic and inflammatory state control effects of RJ and TRF. However, there are a few limitations: we suggest the actions of brown adipose tissue and white fat remodeling in the hypoglycemic effects of RJ via irisin, but we did not assess the underlying pathways and the cascade in which irisin exerts its hypoglycemic effects in detail. Moreover, we could not demonstrate the beneficial metabolic effects of TRF through irisin mediatory action. Since TRF is the mixed isomers of T_3_s and the pure isomers of T_3_s (especially γT_3_) might have different biological activities related to irisin, it is proposed to be investigated in depth.

## 5 CONCLUSION

Royal Jelly (RJ) and TRF improve body weight, glycemic indices, insulin resistance, and inflammation in HFD‐obese rats that underwent CRD. The important new findings of the present research are that RJ remarkably elevates irisin concentrations in HFD‐induced obese rats. Furthermore, irisin is probably the mediator of the promoting efficacy of RJ on glucose hemostasis in the obesity model. It is proposed that the effects of RJ and irisin on obesity‐induced metabolic disorders occur through common pathways, which include inducing thermogenesis through the browning of white adipose tissue, activation of brown adipose tissue, and increasing energy metabolism.

Thus, the recent discovery unravels new areas of research in obesity and its related complications. Further understanding of the signaling molecules and mechanistic pathways through which functional food acts results in favorable appropriate modifications in combating obesity.

## AUTHOR CONTRIBUTIONS

**Naimeh Mesri Alamdari:** Conceptualization (lead); data curation (lead); formal analysis (lead); methodology (lead); writing – original draft (lead); writing – review and editing (lead). **Pardis Irandoost:** Data curation (equal); formal analysis (equal); methodology (equal); writing – review and editing (lead). **Neda Roshanravan:** Writing – original draft (equal); writing – review and editing (equal). **Farzad Najafipour:** Data curation (equal); formal analysis (equal); methodology (equal). **Mohammadreza Vafa:** Project administration (equal); writing – original draft (equal). **Farnaz Farsi:** Methodology (lead); writing – original draft (lead). **Majid Mobasseri:** Data curation (equal); formal analysis (equal); methodology (equal). **Amir Ali Mir Mazhari:** Methodology (lead); visualization (lead). **Halimeh AmirAzad:** Conceptualization (equal); methodology (equal). **Farzad Shidfar:** Project administration (equal); resources (lead); supervision (equal).

## FUNDING INFORMATION

This work was supported by Grant No.: IR. IUMS. REC 96‐02‐27‐31400 from the Iran University of Medical Sciences.

## CONFLICT OF INTEREST STATEMENT

The authors declare no conflicts of interest.

## ETHICS STATEMENT

All animal experimental procedures were authorized via the Ethics Committee of Iran University of Medical Sciences (ethic code: IR.IUMS.FMD. REC 1396.9321324003) and conducted according to the National Institutes of Health Guide for the Care and Use of Laboratory Animals and the ARRIVE guidelines.

## Data Availability

The datasets used and/or analyzed in this investigation are approachable from the corresponding author upon reasonable request.
